# Weekly Variability of an Objective Bradykinesia Score in Parkinson’s Disease—An Observational Longitudinal Study

**DOI:** 10.3390/jcm15093545

**Published:** 2026-05-06

**Authors:** Marcus Dalsgaard Hansen, Filip Bergquist, Trine Hørmann Thomsen

**Affiliations:** 1 Movement Disorder Clinic, Rigshospitalet, 2600 Copenhagen, Denmark; 2 Health Informatics, Department of Public Health, University of Copenhagen, 2200 Copenhagen, Denmark; 3 Department of Clinical Neuroscience, University of Gothenburg, 413 45 Gothenburg, Sweden; filip.bergquist@gu.se; 4 Department of Neurology, Sahlgrenska University Hospital, 413 45 Gothenburg, Sweden; 5 Department of Brain—and Spinal Cord Injuries, Rigshospitalet, 2600 Copenhagen, Denmark; 6 Department of Health Technology, Technical University of Denmark, 2800 Lyngby, Denmark

**Keywords:** Parkinson’s disease, wearables, health technology, bradykinesia, quality of life

## Abstract

**Background:** Parkinson’s Disease is a progressive neurodegenerative disorder characterized by motor and non-motor symptoms. Effective management is critical to improving patient outcomes but is limited by the subjectivity of retrospective assessments of motor symptom burden. The objectives were to describe the weekly variability of objective bradykinesia score (BKS) derived from a wrist-worn device and assess the correlation between BKS variability and the Quality of Life (QoL) of People with Parkinson’s disease (PwP). **Method:** This observational, longitudinal study is part of the overall self-management project, Empower-PD. The study population consists of 80 PwP. Participants wore a wrist-worn accelerometer watch that measures movement during daily activities to evaluate the degree of bradykinesia as the bradykinesia score (BKS). Weekly median BKSs were collected over a 10-week period. Quality of life was assessed with the PDQ-39 questionnaire at baseline and after 10 weeks. Descriptive statistics assessed BKS variation and distribution. Linear and logistic regression models examined correlations between variability in BKS and QoL. **Results:** Results showed that 95% of BKS weekly changes were in a range between −4.3 and +4.4 points, with a central tendency near zero. The distribution of BKSs over time indicated that most participants exhibited stable BKSs. No significant correlation was found between BKS changes and QoL at 10 weeks. **Conclusions:** This study successfully described weekly BKS variations over ten weeks in PwP using objective measurements. The results showed that 95% of all value changes in BKS ranged from −4.3 to 4.4, values which may serve as preliminary distribution-based reference bounds that could inform the design of future studies examining clinically significant changes in BKS.

## 1. Introduction

Parkinson’s disease (PD) is a chronic, neurodegenerative disorder characterized by the gradual loss of dopamine neurons in the brain, leading to motor symptoms like bradykinesia, tremor, and rigidity along with non-motor symptoms (NMS) [1]. Bradykinesia specifically refers to slow movements that display a reduction in automatic movements and/or frequency when movements are repeated. Bradykinesia has a strong negative effect on daily activities [1,2]. Additionally, studies consistently show how the severity of bradykinesia affects patients’ quality of life (QoL) [3,4,5,6], underscoring the importance of understanding its progression and response to medication. Emerging research has further highlighted the role of metabolic disturbances in PD pathogenesis, suggesting that the disease course may be influenced by factors beyond dopaminergic neurodegeneration alone. Notably, variability in glycemic levels and episodic hypoglycemia have been proposed as contributors to disease progression and symptom fluctuation in Parkinsonian syndromes, underscoring the complexity of the underlying pathophysiology [7].

Bradykinesia is the main symptom target that is addressed with current treatments, and information about the occurrence and severity of bradykinesia is therefore important for improving outcomes for People with Parkinson’s disease (PwP). However, previous studies examining the relationship between bradykinesia and QoL have primarily relied on self-reported questionnaires to estimate bradykinesia and severity of motor symptom burden as well as QoL [3,6]. The use of self-reported data is associated with various forms of bias. It has been observed that patients’ responses are often influenced by their recent experiences, particularly the events of the preceding days [8,9], providing a substantial risk of report bias as well as confirmation bias, where individuals tend to report the data that they know their neurologist or PD nurse would like to hear [8]. Several studies have highlighted the advantages of employing objective data collection methods in the treatment of PwP. These benefits include reducing the risk of reporting bias, optimizing individual treatment plans, and enhancing the diagnostic process [9,10,11,12,13].

In recent years, wearable technology has been introduced for the remote and objective collection of abnormal movement data. Parkinson KinetiGraph^TM^ (PKG) is a validated wearable, a wristwatch, that measures PD-relevant movement characteristics. The PKG can support healthcare providers as a complement to better symptom description [14]. Furthermore, it enhances self-understanding of the condition and patients’ illness perception [14]. In summary, wearable technology provides objective data, which can help explain the patient’s condition and bring benefit to both patients and clinicians [14].

Numerous investigations have explored motor symptoms of PD, such as bradykinesia, focusing on aspects including the efficacy of medications, the benefits of specific training regimens, and their overall impact on the QoL of PwP. A study by Löhle et al. [15] specifically examined the temporal distribution of motor symptom changes based on objectively obtained data, also using PKG watches. The study concluded that PKG-based metrics offer a granular depiction of BK’s temporal dynamics. Another study from 2022 [16] investigated the waning duration of the levodopa effect over time and the resulting fluctuations in bradykinesia using sensor data. One notable finding was the impact of these fluctuations on patients’ QoL scores. The authors emphasize the importance of understanding the nature of these fluctuations [16]. Continuous monitoring over an extended period, as conducted in this study, has frequently been recommended in the existing literature as a priority for future research [8,17,18].

As outlined above, previous studies have been short-term, and there are no prior studies with a longitudinal design of comparable scale to this study that describe variations in objective movement over longer time periods that do not strictly reflect daily dosing regimens. A better understanding of the temporal variations over timescales of weeks to months would be useful to determine what the threshold of significant change is. Whether such knowledge could ultimately support automated monitoring systems with threshold-triggered clinical contact pathways represents an interesting hypothesis that warrants investigation in future interventional studies.

Therefore, the primary objective of this study is to describe the variations and distribution of weekly median BK scores (BKSs) within a cohort of 80 PwP, utilizing objective measurements. Secondly, the study aims to establish threshold values for presumed BKS changes and to investigate the potential correlation between changes in BKS and QoL.

## 2. Methods

### 2.1. Study Design

This study uses an observational longitudinal design to describe the weekly changes in BK. It is part of the self-management project “EMPOWER-PD”, where participants have been educated and guided in integrating disease-related knowledge, insights from subjective data from the PKG, and mental strategies into self-management of everyday life with PD [19]. The self-management project aimed to develop, test, and evaluate a co-designed self-management program for individuals with PD.

### 2.2. Recruitment and Participants

To be eligible for this study, measurements of BKS during 6 non-continuous 6-day measurement periods within a 10-week period needed to be obtained and reported in the PKG data portal. The 10-week timeframe was chosen to align with the duration of the EMPOWER-PD self-management program [20], ensuring that BKS data collection was concurrent with the intervention period. Each participant therefore contributed six discrete measurement windows, each spanning six consecutive days of accelerometer wear, distributed across the 10-week period. To ensure sufficient temporal coverage while allowing for practical flexibility in device return and reapplication, no more than 10 days were permitted between any two measurement periods. This design was intended to capture meaningful longitudinal variation in BKS while remaining feasible within the clinical and logistical constraints of the study. From these six measurement periods, five consecutive pairwise differences were derived per participant—each representing the change in median BKS from one recording period to the next—thereby forming the basis for the weekly variability analyses conducted in this study. The EMPOWER-PD study recruited participants from October 2022 to January 2024 through the Movement Disorder Clinic (MDC) at Rigshospitalet, Copenhagen University Hospital Bispebjerg, Frederiksberg University Hospital, Copenhagen, Denmark, a private neurologist, a physiotherapist clinic, and a specialized rehabilitation center (Sano) in Capitol Region. A total of 96 candidates were screened based on predefined inclusion and exclusion criteria [20].

**Figure 1:**
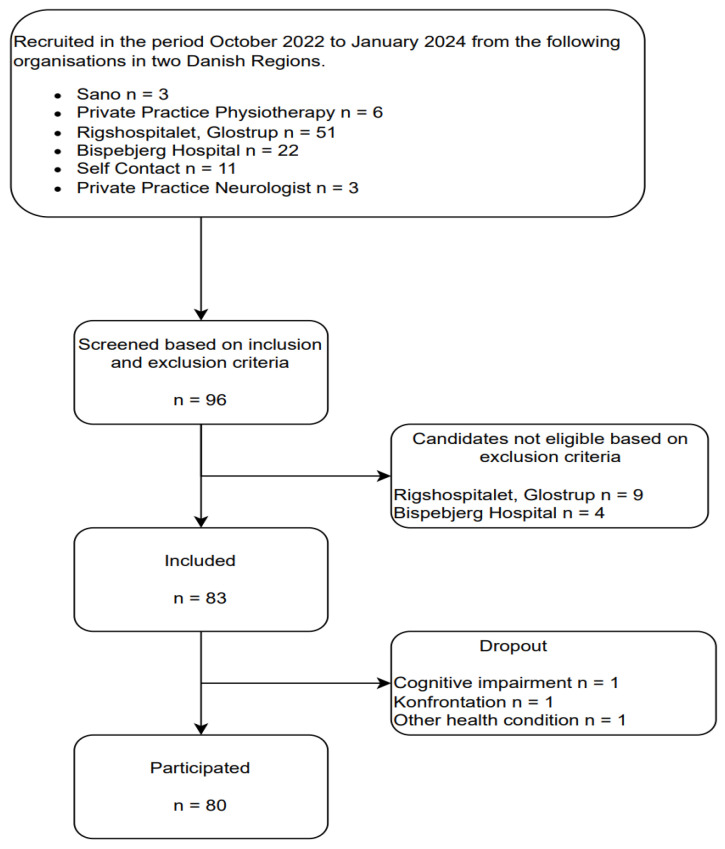
Flowchart of the recruitment and inclusion process.

Eligibility criteria included diagnosis of idiopathic PD, Hoehn & Yahr stage of disease [1,2,3], the ability to provide informed consent, and the physical capacity to participate in the self-management program. Exclusion criteria were significant cognitive impairments (based on a Montreal Cognitive Assessment score < 24) and comorbidities that would interfere with participation [20]. This resulted in a final cohort of 80 participants who successfully completed the study (Figure 1).

### 2.3. Data Collection

BKSs were obtained using the PKG wrist-worn logger, which records continuously over multiple days during routine activities [12]. The monitors were worn on the most affected wrist side. 

The PKG computes a BKS and a dyskinesia score for every 2 min epoch; summary metrics are derived as medians across epochs collected between 09:00 and 18:00 over ≥6 recording days [12]. The algorithmic derivation of BKS and its clinical validation against established rating scales have been described in detail elsewhere [12,21]. In line with these sources, sleep and daytime sleepiness were identified from episodes of immobility using specific immobility/sleep detection methods, rather than by assigning fixed BKS bands (e.g., “BKS 80–110”) to sleep-like states [12]. Interpretation of bradykinesia severity in the PKG literature primarily relies on distributional measures relative to controls (e.g., the proportion of epochs with BKSs above the 50th percentile of controls, BKS > 50) and aggregated indices such as the median BKS, not on absolute cut-off points such as 40, 80, or 110 [12,21].

The QoL score was obtained using a self-reported questionnaire, the Parkinson’s Disease Questionnaire (PDQ-39) [22]. PDQ-39 is the most widely used disease-specific measure of health-related quality of life in PwP. The domains include mobility, activities of daily living, emotional well-being, stigma, social support, cognitions, communications and bodily discomfort [23]. The participants filled out PDQ-39 ratings at baseline and after 10 weeks, providing an opportunity to identify a longitudinal change in their scores over 10 weeks. Data was entered in EasyTrial, a 2-way factor authentication data storage system, by a research assistant at the MDC, Rigshospitalet, Denmark.

BKS data was collected from the PKG portal and QoL data from the EasyTrial database in March 2024 after the final group of participants out of 80 completed the 8-week self-management program.

### 2.4. Statistical Methods and Analysis

The statistical work was conducted using the software environment R in R-studio version 2023.06.1+524. Descriptive statistics were conducted to establish the weekly variations and distribution of participants’ median BKS. To analyze the correlation between BKS and QoL, linear and logistic regression were used as statistical models, and the Welch T-test was used as a statistical test method for group comparison.

To answer the primary objective, a variable termed Weekly BKS Change (WBKSC) has been calculated, where the dataset was converted to long format, so each participant had 5 observations within this continuous variable. The dataset was restructured from wide to long format, whereby each participant contributed five individual observations corresponding to the consecutive weekly score differences. A custom function, calc_stats(), was applied to compute descriptive statistics for the variable, including median, mean, standard deviation, minimum, maximum, first and third quartiles, as well as the 2.5th and 97.5th percentiles derived from a normal distribution. Missing values were systematically excluded from all calculations (na.rm = TRUE).

Changes in BKS were calculated based on the absolute value (Mean Absolute BKS Change, MABKSC). The variable MABKSC represents the mean absolute change across six measurement weeks for each participant. Initially, weekly differences were calculated between consecutive weeks (week 2 minus week 1, week 3 minus week 2, etc.), yielding five difference variables. Subsequently, the absolute value of each difference was computed, ensuring that both positive and negative fluctuations are treated as genuine deviations rather than offsetting one another. Finally, the mean of the five absolute differences was calculated for each participant using the rowMeans() function. Missing values were handled by excluding them from the computation (na.rm = TRUE).

As an exploratory and supplementary analysis, participants were dichotomized around the cohort median MABKSC into a lower-variability group and a higher-variability group, hereafter referred to as stable and shifting patients, respectively. This stratification was not intended to represent a clinically validated classification but rather to provide a simplified illustration of whether participants at the lower and upper ends of the variability spectrum differed meaningfully in their QoL outcomes. The two groups were compared using the Welch corrected *t*-test. It is acknowledged that this dichotomization simplifies a continuous phenomenon and that the median as a cut-point is inherently arbitrary; the results of this analysis should therefore be interpreted as hypothesis-generating rather than conclusive.

The analysis was found to be robust after validating the four core linear regression assumptions: independence, linearity, normal distribution, and homoscedasticity. Linearity was confirmed through residual plots. Participants were recruited independently via the EMPOWER-PD study [19], such that enrolment procedures were exogenous to, and uninfluenced by, the present analysis. 

However, after reshaping the dataset from wide to long format, the analytic file comprises 400 observations derived from 80 individuals (five repeated measures per participant); accordingly, statistical independence at the observation level is attenuated. It should be noted that the primary objective of this study was descriptive in nature, aiming to characterize the distributional properties of weekly BKS changes across the cohort rather than to model within-subject trajectories or causal associations. For this purpose, the pooling of repeated observations into a single distribution is a recognized approach, as the estimation of population-level percentile boundaries, such as the 2.5th and 97.5th percentiles used to define the ±4.3–4.4 range, does not inherently require statistical independence between observations [24]. The non-independence of repeated measures is of primary concern when the goal is hypothesis testing or effect estimation at the individual level, where it can artificially inflate degrees of freedom and narrow confidence intervals. In the secondary regression analyses examining the association between BKS variability and QoL, this limitation is acknowledged, and the results should accordingly be interpreted as exploratory and hypothesis-generating rather than confirmatory. A mixed-effects modelling framework would be the preferred approach in future studies with a larger sample, as it would allow for explicit partitioning of within- and between-subject variance and yield more robust inferential estimates. The current analytical choices were made pragmatically given the sample size and the primarily descriptive scope of the study.

Normality of residuals was assessed using Q-Q plots, while homoscedasticity was evaluated via residual plots. Although some Q-Q plots showed small deviations at the ends, which could suggest possible outliers and non-normality, linear regression was still deemed appropriate.

## 3. Results

The study population consists of 80 PwP from the EMPOWER-PD study [20], and data from these were included in the analysis. Table 1 displays descriptive statistics of participant demographics. Some key demographics show gender distribution percentages of 53.8% men and 46.2% women. Participants had a mean age of 64.3 years (range: 43–86) and an average disease duration of 6.0 years (range: 1–23).

At baseline, the mean BKS was 28.4 (SD 6.2, range 14.0–41.5), the mean Dyskinesia score (DKS) was 1.9 (SD 2.6, range 0.1–19.7), and the mean PTI was 8.5% (SD 6.1, range 0.5–32.4). These values indicate, on average, mild to moderate bradykinesia, low dyskinesia burden, and relatively limited immobility, although with notable inter-individual variability.

Disease severity assessed using the Hoehn & Yahr scale showed that 36.3% were in stage 1, 42.5% in stage 2, and 21.2% in stage 3. Cognitive function, measured by the MoCA score, had a mean of 27.8 (range: 24–30). Education levels varied, with 43.7% having higher education and 28.8% holding graduate degrees. Co-morbidity analysis revealed that 52.5% had no additional chronic conditions, 31.3% had 1–2, and 16.2% had more than two, with 6.3% requiring home nursing. Table 1 also shows categorically which comorbidities the participants have.

Overall, the study population consisted of a relatively well-functioning group of PwP with preserved cognitive abilities and diverse educational and health backgrounds.

**Figure 2:**
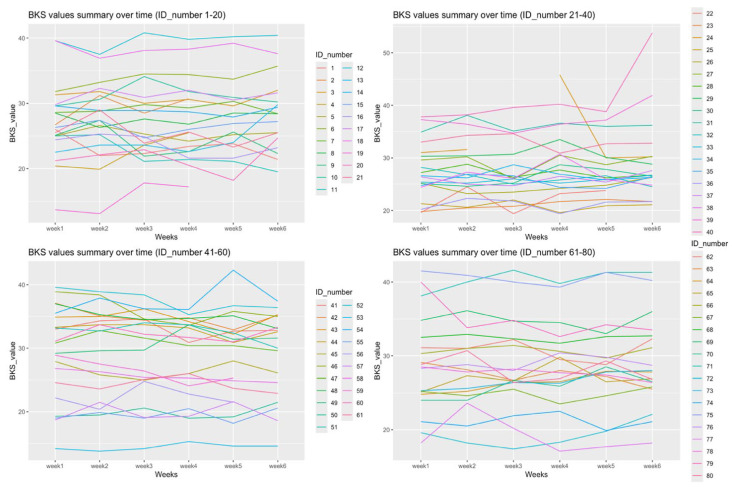
Spaghetti plot of participants’ individual BK-values across 6 weeks.

Figure 2 displays each participant’s weekly median BKS over the 6-week period. Most participants did not display large fluctuations in weekly median BKS, except for a few participants who had larger deviations in single weeks. The outlier median BKS were not associated with low use of the device, as suggested by off-wrist detection.

**Figure 3:**
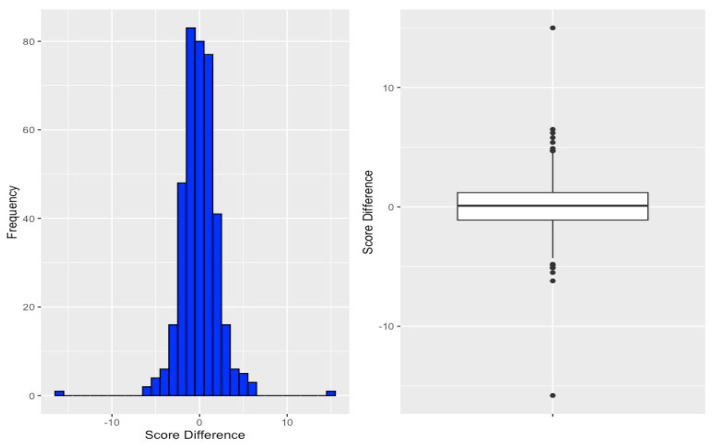
Boxplot and histogram for weekly BKS change distribution.

The distribution of BKS differences is mostly compressed and compact, which is visualized in Figure 3, with descriptive statistics presented in Table 2. The mean and median score is 0.1 with a standard deviation (SD) of 2.2. The 2.5th and 97.5th percentiles, at −4.3 and 4.4, respectively, left 95% of all values in the range between −4.3 and 4.4. There were two outlier weekly BKS change values at −15.8 and 15.

A linear regression analysis was performed to evaluate a possible correlation between the mean BKS values of the preceding six non-continuous 6-day measurement periods within the 10-week period and the QoL measure reflecting the 10-week period. Results from the linear regressions to check the correlation between weekly changes in BKS and patients’ QoL score after 10 weeks revealed no significant effect (*p* = 0.90, 95% CI: −1.68 to 1.93). There was a strong correlation between QoL at baseline and at week 10 (*p* = < 0.001, 95% CI: 0.57 to 0.72). The linear regression model revealed that MABKSC accounted for a negligible proportion of the variance in QoL scores (R^2^ = 0.006), indicating an effect size considerably below the conventional threshold for a small effect.

A predictor of percent change in QoL was also examined. Linear regression yielded no significant effect (*p* = 0.60). An adjusted model adding years with PD showed similar results (*p* = 0.64). Furthermore, linear regression of percent QoL change on normalized BKS changes yielded a coefficient but was not statistically significant (*p* = 0.60).

The exploratory Welch *t*-test comparing the stable and shifting patient groups showed no significant difference in QoL at the end of the study period (*p* = 0.14, 95% CI: −11.82 to 1.77). This finding is consistent with the regression results.

## 4. Discussion

We were able to describe the variability in median change in BKS and demonstrate that 95% of the changes in BKS over a period of 10 weeks were within the range [−4.3, 4.4]. This range was derived empirically from the observed distribution of weekly BKS changes, where the 2.5th and 97.5th percentiles were identified as −4.3 and 4.4, respectively. Under the assumption of an approximately normal distribution, these percentile boundaries define the interval within which 95% of all observed changes fall, meaning that changes exceeding ±4.3 points lie in the outermost 5% of the distribution and can thus be considered statistically atypical relative to the natural week-to-week variation observed in this cohort. There was no significant correlation between QoL scores after 10 weeks and changes in BKS.

The BKS curves indicate that scores remained relatively stable for most participants over the 10-week period, with only a minority showing marked week-to-week deviations. The limited variability in BKSs may be related to the relatively short mean disease duration in this cohort, which aligns with previous findings that motor fluctuations tend to emerge in the moderate to late stages of Parkinson’s disease [25]. The study population had a mean disease duration of six years and was restricted to Hoehn & Yahr stages 1–3, meaning that patients with more advanced disease, pronounced motor fluctuations, cognitive decline, or complex polypharmacy regimens were not represented. This is an important consideration when interpreting the observed stability of BKSs, as variability patterns may differ substantially in patients with more severe disease. It is worth noting that this relationship is likely non-linear; while motor fluctuations may be more pronounced in moderate-to-advanced stages of PD, patients at the latest stages of the disease tend to exhibit consistently poor motor outcomes with reduced week-to-week variability, as bradykinesia becomes more stable and less responsive to treatment [26]. The findings of this study should therefore be understood as primarily applicable to individuals in the early to mid stages of PD, and generalization beyond this population is not supported by the present data. It would be of considerable interest to conduct a comparable study in a cohort with longer disease duration and more advanced motor complications.

Previous research has demonstrated that fluctuations in bradykinesia can affect PwP’s QoL [4], and pharmacological treatment itself plays a central role in modulating these fluctuations, as long-term levodopa therapy has been associated with the development of motor complications that can further compromise QoL [4]. The analysis from our study revealed no significant impact of weekly median BKS changes on QoL after the 10-week period, suggesting that week-to-week variations in BKS may have little influence on perceived QoL. While weekly fluctuations may not significantly affect QoL, a sustained decrease in BKS over a longer period may have a more substantial impact. Within this specific cohort and observation window, weekly changes in median BKS exceeding ±4.3 points fall outside the 95% empirical distribution of observed week-to-week variation, and may therefore represent a preliminary reference bound for what constitutes an atypical fluctuation. However, this should not be interpreted as a validated clinical threshold, as the present data reflect the distributional properties of one cohort and have not been tested in the context of treatment response or clinical decision-making. As many patients have median BKS around 26 [27], a change of median BKS of 4.3 is a change of roughly 15%. It remains to be determined what the minimal clinically important change in median BKS is, and the current findings may serve as preliminary reference values that could inform the design of future studies, where changes in median BKS, for example, in the context of treatment initiation or dose reduction due to adverse effects, could be examined in relation to patient-perceived symptom burden. Whether such values could eventually inform clinical decision thresholds, including when to modify treatment or initiate patient contact, remains to be tested prospectively and cannot be concluded from the present dataset alone.

The absence of a significant association between weekly BKS changes and QoL in this study is perhaps not unexpected when considered in light of the multidimensional nature of QoL in PD. The PDQ-39 captures a broad spectrum of domains, including emotional well-being, social function, cognition, and bodily discomfort, all of which are substantially influenced by non-motor symptoms rather than motor fluctuations alone [6]. Indeed, Gökçal et al. [6] demonstrated that non-motor symptoms, particularly anxiety and bladder and sexual dysfunction, are among the strongest self-reported determinants of poor QoL in PwP, suggesting that a narrow motor metric such as weekly median BKS variability may simply not be expected to track changes in PDQ-39 scores in a cohort where non-motor burden was not directly measured or controlled for. This represents a fundamental construct mismatch between the exposure variable and the outcome, and the negative result should therefore be interpreted as reflecting this mismatch rather than an absence of clinical relevance of BKS as a measure. Furthermore, motor symptom progression in PD is inherently gradual, and meaningful changes in BKSs that could plausibly translate into perceived QoL differences may require a considerably longer observational window to manifest. In a relatively stable, early-to-mid-stage cohort observed over only 10 weeks, the absence of a significant correlation is consistent with what the study design could reasonably be expected to detect, and an extended longitudinal design including the assessment of non-motor symptom burden would be better positioned to disentangle these relationships. For instance, stratifying the sample based on Hoehn & Yahr scores could provide valuable insight into whether disease severity has a differential impact on the observed outcomes. However, the current sample size is insufficient to yield statistically reliable results when divided into multiple subsets, thereby limiting the ability to draw meaningful comparisons across disease stages.

Continuous tracking of BK, rather than infrequent clinical assessments, may lead to better treatment strategies and patient empowerment. To implement this, it is necessary to establish thresholds for abnormal changes in objective movement measures over the natural variation that comes from regular daily activities. The results from this study may serve as a starting point for future research aimed at better understanding weekly bradykinesia variability, which in turn could inform the development of more individualized treatment strategies, though direct application to clinical decision-making requires further validation. Insights from the EMPOWER study [20] indicated that some patients, through verbal feedback, reported experiencing stress related to difficulties in interpreting data and handling technical issues. This highlights the importance of providing adequate education and support to ensure that patients can fully benefit from wearable technologies. Beyond pharmacological treatment, the BKS may also hold potential as an outcome measure for evaluating the effects of non-pharmacological interventions. As part of the self-management program in the EMPOWER-PD study [20], participants were encouraged to set personal health goals targeting modifiable lifestyle factors such as physical activity, sleep quality, and the management of symptoms like constipation. In select cases, improvements in these areas appeared to coincide with favorable changes in BKS, suggesting that objective bradykinesia monitoring may be a feasible tool for assessing the impact of non-pharmacological approaches on motor function. While these observations are preliminary and based on individual cases, they point to a broader applicability of continuous BKS tracking beyond medication titration. PwP may gain peace of mind and a better understanding of their condition by having the opportunity to monitor their values with information about how much they can be expected to vary over time. The study by Thomsen et al. [20] demonstrates that increased patient knowledge enhances their ability to take strategic action, providing them with more effective tools for managing their condition. Furthermore, median BKS may serve as a valuable indicator for future clinical trials. The intention of the present study is precisely to identify threshold values that can be applied as eligibility criteria for study participation, as well as for the stratification of study populations in each research context. Objective measurement technologies, such as the PKG watch, remain relatively novel, and considerable knowledge is yet to be established in this area. The present study has contributed to this by providing median bradykinesia score values, which may serve as indicators for, amongst other things, the development of disease profiles.

Several limitations of this study warrant critical consideration. The sample size of 80 patients, while offering preliminary insights, substantially limits the generalizability of the findings to the broader PD population. The study population was skewed towards earlier disease stages, with a mean disease duration of six years and no inclusion of patients with Hoehn & Yahr stages 4 or 5, meaning that patients with more advanced disease, pronounced motor fluctuations, cognitive decline, or complex polypharmacy regimens were not represented. This is an important consideration, as variability patterns may differ substantially in patients with more severe disease, where the relationship between bradykinesia and weekly fluctuation could be considerably more pronounced. The findings should therefore be understood as primarily applicable to individuals in the early to mid stages of PD, and generalization beyond this population is not supported by the present data. Furthermore, the cohort was recruited from specialized movement disorder clinics, introducing a potential selection bias towards better-functioning, more health-literate patients, which may inflate the stability of the observed BKS values. Nevertheless, this is one of the largest datasets where long-term continuous objective measurements have been reported.

From an analytical standpoint, a significant methodological limitation must be acknowledged. The use of approximately 400 observations derived from 80 individuals, each contributing five repeated measures, fundamentally violates the assumption of statistical independence that underlies the linear regression models applied. This inflation of the effective sample size risks producing artificially narrow confidence intervals and *p*-values, thereby potentially overstating the precision of the reported estimates. While the regression results were interpreted cautiously, the absence of mixed-effects models or other approaches that explicitly account for within-subject correlation represents a meaningful weakness that tempers the strength of the statistical conclusions. For the preliminary reference bounds identified in this study to progress towards any form of clinical application, future studies will need to adopt a mixed-effects modelling framework, which would allow for explicit partitioning of within- and between-subject variance, properly account for the repeated-measures structure of the data, and yield inferential estimates robust enough to support clinically meaningful interpretation.

## 5. Conclusions

This study successfully addressed its primary objective by describing the weekly variations in BKS over a ten-week period in a cohort of PwP using objective accelerometer measurements. The results showed that 95% of all observed weekly changes in BKS fell within the range of −4.3 to 4.4, providing preliminary distribution-based reference bounds for week-to-week variability in this relatively homogeneous, early-to-mid-stage cohort. These values should be regarded as hypothesis-generating rather than validated clinical thresholds, as the present data reflect the distributional properties of one specific cohort and have not been tested in the context of clinical decision-making or treatment response. No statistically significant change in BKS was observed over the 10-week period, and no significant correlation was identified between BKS changes and QoL at 10 weeks. The absence of this association is likely to reflect, at least in part, a construct mismatch between a narrow motor metric and a multidimensional QoL measure, rather than an absence of clinical relevance of BKS monitoring. Future studies involving larger, more diverse cohorts—including patients with more advanced disease, greater motor fluctuations, and higher non-motor symptom burden—are needed to validate these preliminary reference bounds, determine clinically meaningful thresholds for BKS changes, and examine whether objective bradykinesia monitoring can inform individualized treatment decisions.

## Figures and Tables

**Table 1:** Descriptive statistics of the participants’ demographic and clinical data. * Hypertension, diabetes, cardiovascular disease, hypercholesterolemia; ** arthritis, osteoporosis, musculoskeletal injuries; *** depression, anxiety and other psychiatric conditions.

	Mean/SD	Range
Age	64.3/9.8	43–86
Disease duration (years)	6.0/4.2	1–23
MoCA-score	27.8/1.5	24–30
Bradykinesia score (BKS) baseline	28.4/6.2	14–41.5
Dyskinesia score (DKS) baseline	1.9/2.6	0.1–19.7
Percent Time Immobility (PTI) baseline	8.51/6.1	0.5–32.4
Medication LED-score	578/340	40–1898
Gender	N	%
Men	43	53.8
Women	37	46.2
Hoehn & Yahr score		
1	29	36.3
2	34	42.5
3	17	21.2
Working status		
Yes	16	20.0
Yes, flex job	11	13.8
Early retirement	25	31.2
Retired	28	35.0
Education		
7 or fewer years in school (middle school)	4	5.0
8–11 years in school (high school)	18	22.5
Graduate degree (master’s degree or similar)	23	28.8
Higher education (postgraduate, etc.)	35	43.7
Home nursing		
Yes	2	6.3
No	75	93.7
Independence in ADLs		
Yes	66	82.5
No	14	17.5
Co-morbidity		
Yes (1–2 chronic conditions)	25	31.3
No	42	52.5
More than 2 chronic conditions	13	16.2
Co-morbidity categories		
Cardiovascular and metabolic disorders *	76	
Musculoskeletal disorders **	35	
Neuropsychiatric disorders ***	43	

**Table 2:** Descriptive statistics for the collected score difference.

	Median	Mean	SD	Min. Value	Max. Value	1st Q.	3rd Q.	2.5%	97.5%
Weekly BKS Change	0.1	0.1	2.2	−15.8	15	−1.1	1.2	−4.3	4.4

## Data Availability

The original contributions presented in this study are included in the article. Further inquiries can be directed to the corresponding authors.
